# Supplementary material for: Influences of Substrate Grain Size on the Burrowing Behavior of Juvenile Meretrix meretrix
Source: Animals (Basel). 2022 Aug 16;12(16):2094. doi: 10.3390/ani12162094 (PMC9405232; doi:10.3390/ani12162094)
Supplement: Supplementary file 1 [file animals-12-02094-s001.zip › animals-1830768-supplementary.pdf]

animals

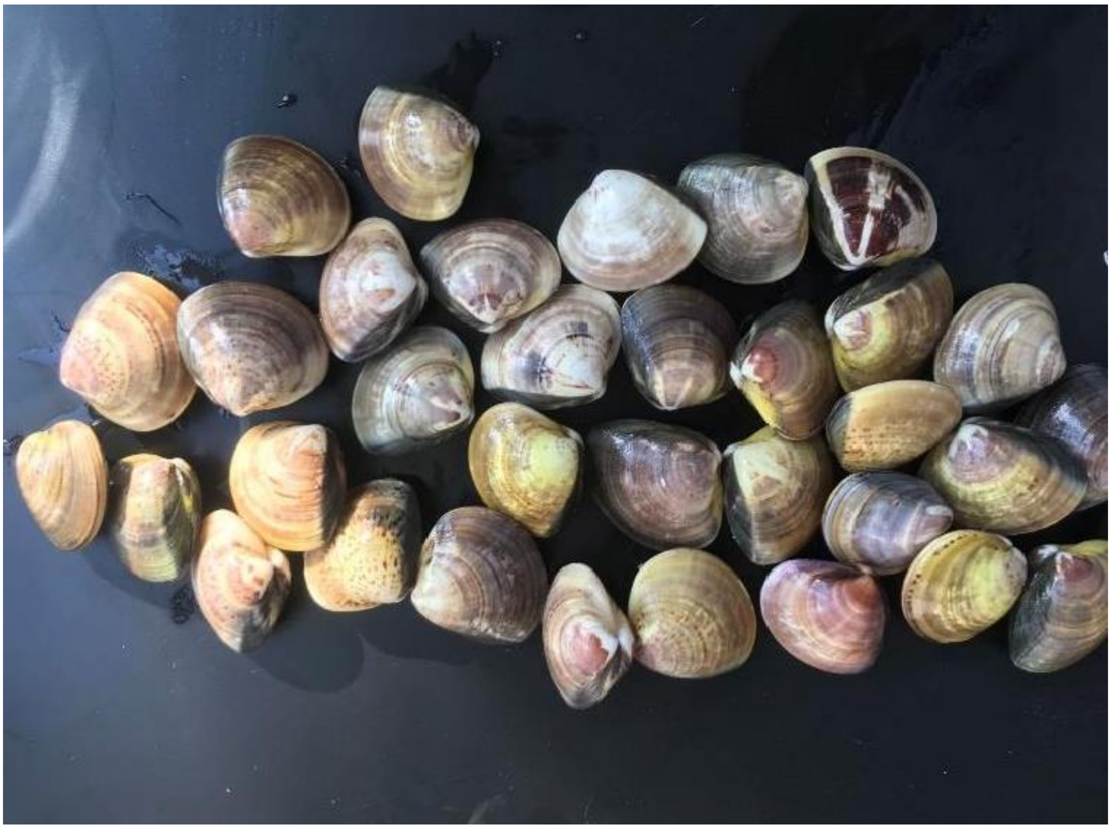

Figure S1. The photo of *Meretrix meretrix.*

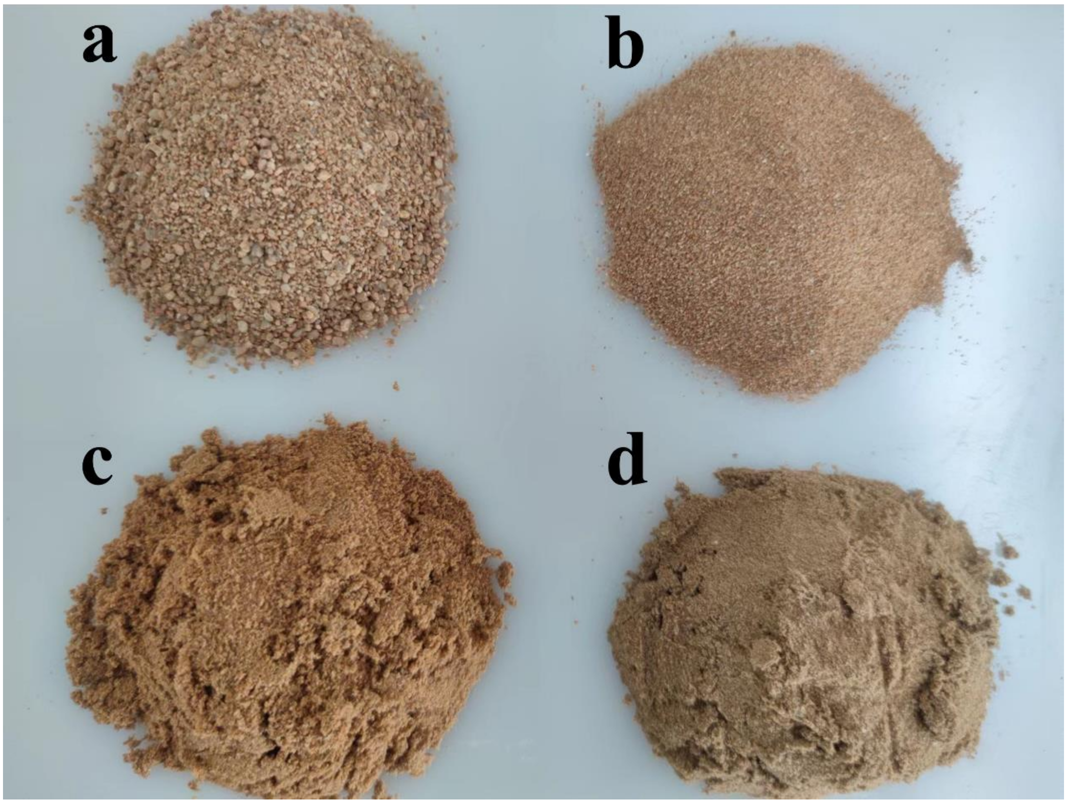

Figure S2. Four substrates. (**a**), coarse sand; (**b**), medium sand; (**c**), fine sand; and (**d**), natural substrate.

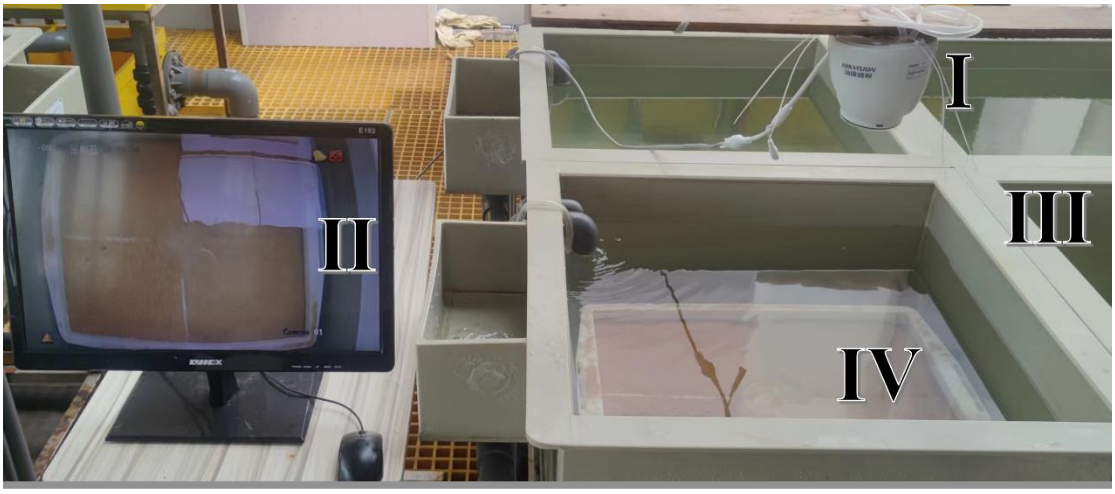

Figure S3. The entire experimental apparatus. Unit I is a camera (4 MP), unit II is a monitor (546.1 mm), unit III is the recirculating water system, unit IV is the experimental device.
